# Complete Genome Sequence of the Avian Pathogenic *Escherichia coli* Strain APEC O78

**DOI:** 10.1128/genomeA.00026-13

**Published:** 2013-03-21

**Authors:** Paul Mangiamele, Bryon Nicholson, Yvonne Wannemuehler, Torsten Seemann, Catherine M. Logue, Ganwu Li, Kelly A. Tivendale, Lisa K. Nolan

**Affiliations:** a Department of Veterinary Microbiology and Preventive Medicine, College of Veterinary Medicine, VMRI 2, Iowa State University, Ames, Iowa, USA; b Victorian Bioinformatics Consortium, Monash University, Clayton, Victoria, Australia; c Veterinary Science, the University of Melbourne, Parkville, Victoria, Australia

## Abstract

Colibacillosis, caused by avian pathogenic *Escherichia coli* (APEC), is a significant disease, causing extensive animal and financial losses globally. Because of the significance of this disease, more knowledge is needed regarding APEC's mechanisms of virulence. Here, we present the fully closed genome sequence of a typical avian pathogenic *E. coli* strain belonging to the serogroup O78.

## GENOME ANNOUNCEMENT

Colibacillosis, caused by avian pathogenic *Escherichia coli* (APEC), is one of the most significant infectious diseases affecting poultry (1–7). Poultry colibacillosis takes many forms, with systemic forms occurring most often (2). Collectively, these diseases result in annual multimillion-dollar losses due to mortality, decreased production, and condemnations (1, 3, 5). Indeed, colibacillosis poses a profound threat to one of humankind’s cheapest sources of high-quality animal protein. Despite the importance of this disease, the mechanisms of APEC virulence remain largely unknown. Studies into APEC pathogenesis would be enhanced by public access to high-quality genomic sequences. To date three APEC sequences are publically available. The sequence of APEC O1, an O1:K1:H7 strain isolated from the lung of a turkey, is fully closed (9). Obtained from gelatinous edema lesions from a laying hen, a draft sequence of a Brazilian APEC strain, SCI-07, a member of the O nontypeable:H31 serotype, is in 68 contigs (10), and a sequence of an O78 strain (χ7122) was recently released in 12 contigs (11). Here, we describe a fully closed and annotated sequence of an O78 strain with the idea that fully closed sequences representative of the most commonly isolated APEC serogroups, such as O1 and O78 strains, are needed to adequately support future colibacillosis research (1).

APEC O78 is an O78 strain isolated from the lung of a turkey clinically diagnosed with colibacillosis. Genomic sequencing was performed using complementary sequencing technologies, combining results obtained with a Roche/454 FLX genome sequencer (GS) instrument and an Illumina Hi-Seq 2000. The following datasets were used in the final assembly: (i) GS-FLX, with 590,773 shotgun reads totaling 237 Mb (~49-fold coverage); (ii) GS-FLX 8-kb mate-pair library with 474,583 shotgun reads totaling 168 Mb (~35-fold coverage) of which 330,857 were paired; and (iii) Illumina 100 bp paired-end library with 27,389,600 reads totaling 2,587 Mb (~539-fold coverage). Both 454-read sets were assembled *de novo* using Newbler 2.6 (Roche), and Illumina reads were assembled separately with Velvet 1.1 (8) and Illumina’s ELANDv2e assembler. The genome was closed using 454 assemblies as a “reference” sequence and the Illumina data to add depth, correct errors, and close gaps. Whole-genome optical mapping (OpGen, Gaithersburg, MD) was used to validate scaffolds and contig order. The assembly was confirmed using PCR and Sanger sequencing and validated by consistency of paired-end evidence from 454 and Illumina reads.

Annotation was automated using the NCBI Prokaryotic Genomes Automatic Annotation Pipeline (PGAAP). The final version was checked against the previously completed Prokka 1.5.2 annotation.

The assembled genome consists of a single chromosome (4,798,435 bp; 50.68% GC content) and two plasmids, one 217.830 kb and the other 113.260 kb. The chromosome contains 4,696 protein-encoding genes, 88 tRNA-carrying genes, and 19 rRNA-carrying operons. The chromosome of APEC O78 is smaller than many other fully sequenced extraintestinal pathogenic *E*. coli (ExPEC) genomes, and its chromosomal structure appears different from those of other ExPEC genomes. Assessment of the implications of these differences is ongoing, but the addition of a fully closed genomic sequence of one of the commonly occurring serogroups among APEC significantly contributes to the toolset that can be used in studies of APEC pathogenesis and colibacillosis control.

### Nucleotide sequence accession number. 

Complete sequences of APEC O78 have been deposited in GenBank under accession number CP004009.
